# ﻿*Dipterisshenzhenensis*, a new endangered species of Dipteridaceae from Shenzhen, southern China

**DOI:** 10.3897/phytokeys.186.73739

**Published:** 2021-12-09

**Authors:** Zuo-Ying Wei, Yu-Feng Gu, Zeng-Qiang Xia, Li-Jun Chen, Ting Wang, Shou-Zhou Zhang, Guo-Hua Zhao, Jian-Bing Chen, Jian-Guo Cao, Yue-Hong Yan

**Affiliations:** 1 Key Laboratory of National Forestry and Grassland Administration for Orchid Conservation and Utilization, The National Orchid Conservation Center of China and The Orchid Conservation and Research Center of Shenzhen, Shenzhen 518114, China Shanghai Normal University Shanghai China; 2 College of Life Sciences, Shanghai Normal University, Shanghai 201602, China Key Laboratory of National Forestry and Grassland Administration for Orchid Conservation and Utilization, The National Orchid Conservation Center of China and The Orchid Conservation and Research Center of Shenzhen Shenzhen China; 3 Life Science and Technology College, Harbin Normal University, Key Laboratory of Plant Biology in Colleges of Heilongjiang Province, Harbin 150025, China Harbin Normal University Harbin China; 4 CAS Center for Excellence in Molecular Plant Sciences, Shanghai Institute of Plant Physiology and Ecology, Chinese Academy of Sciences, 300 Fenglin Road, Shanghai 200032, China Shanghai Institute of Plant Physiology and Ecology, Chinese Academy of Sciences Shanghai China; 5 College of Biodiversity Conservation, Southwest, Forestry University, Kunming 650224, China Southwest Forestry University Shenzhen China; 6 Shenzhen Key Laboratory of Southern Subtropical Plant Diversity, Fairy Lake Botanical Garden, Shenzhen Fairy Lake Botanical Garden, Shenzhen & Chinese Academy of Sciences Shenzhen China; 7 Chinese Academy of Sciences, Shenzhen 518004, China Shanghai Normal University, Shanghai China

**Keywords:** fern, Gleicheniales, morphology, phylogeny, quantity traits, taxonomy

## Abstract

*Dipterisshenzhenensis*, a new species of ferns from Shenzhen, Guangdong, southern China, is identified and described. It closely resembles *D.chinensis* but possesses several unique traits, such as long rhizome scales, castaneous stipe, and abaxially pale fronds with two fan-shaped fronds connected by a broad wing. Molecular evidence showed that *D.shenzhenensis* is allied to *D.conjugata*, whereas it has morphologically significant differences (*P* < 0.05) on the basis of quantitative trait statistical analysis. Overall, the morphological evidence, taken together with the result of cpDNA indicated that *D.shenzhenensis* is a distinct species.

## ﻿Introduction

*Dipteris* Reinw. is one of two genera in Dipteridaceae (Zhang et al. 2013; PPG I 2016), and is considered as an early-diverging leptosporangiate fern lineage related to the Gleicheniaceae (Schuettpelz and Pryer 2007; Lehtonen 2011). The genus has rare components consisting of about eight species, and is restricted to Indo-Malaysian Islands, including north-eastern India, southern China, and from the southern Ryukyus to northeast Queensland (Australia) and Fiji (Kramer 1990; Zhou et al. 2016; Choo and Escapa 2018; Zhang et al. 2013). The morphology of *Dipteris* is characterized by having long creeping rhizomes and fan-shaped fronds possessing elaborately anastomosing veins with free veinlets in the areoles (Bomfleur and Kerp 2010; Tidwell and Ash 1994).

In August 2020, during botanical research on Mt. Qiniangshan in Shenzhen, Guangdong, southern China, a unique species of *Dipteris* was documented on rocks in evergreen broad-leaf forest. The species is so similar to *D.chinensis* Christ that it has always been interpreted as the latter (Yan 2017). Upon closer carefully specimen identification and comparison with other species in *Dipteris*, we found that this unknown species possesses several unique characteristics, the most striking of which is awfully long rhizome scales. Furthermore, we constructed the molecular phylogeny of *Dipteris* to obtain a phylogenetic insight into the species. The morphological evidence taken together with the result of cpDNA validated it as a new species.

## ﻿Materials and methods

### ﻿Morphological analyses

The features of rhizome scales were obtained using a Leica M205A dissecting microscope. Morphology of spores was observed with Phenom Pro scanning electron microscope after being sputter-coated with gold. Measurements were made from mature and intact specimens. For length and width of lobes, each specimen was measured six times using ImageJ software (Collins 2007), followed by taking an average. All images of specimens were provided by the National Specimen Information Infrastructure (http://www.nsii.org.cn), Global Biodiversity Information Facility (https://www.gbif.org/), and JSTOR (https://plants.jstor.org/). Voucher specimens were deposited in the National Orchid Conservation Center of China (NOCC) and Shenzhen Fairy Lake Botanical Garden Herbarium (SZG).

### ﻿Phylogenetic analyses

Ten samples, representing five species were used in this study. Apart from the sequences of *Dipterisconjugata* Reinw. (Metzgar et al. 2008) and the outgroup *Cheiropleuriaintegrifolia* (D. C. Eaton ex Hook.) M. Kato, Y. Yatabe, Sahashi & N. Murak. (Schuettpelz and Pryer 2007) were downloaded from GenBank, all other sequences were newly generated (Table S1). Five chloroplast DNA (cpDNA) regions (*rbcL*, *atpA*, *rps4*, *rbcL-accD* and *trnG-trnR*) were extracted, amplified and sequenced following Wei et al. (2021). Primers used for polymerase chain reaction (PCR) amplification and sequencing are shown in Table 1. All sequences newly generated in this study were deposited in GenBank (see Table S1 for accession numbers). The cpDNA sequences were assembled and edited using SeqMan v.7.1.0 (DNASTAR, USA), then aligned using MEGA v.7.0 (Kumar et al. 2016). Alignments of five genes were concatenated using PhyloSuite (Zhang et al. 2020), and best nucleotide substitution model (Table 2) was used on the basis of Akaike Information Criterion with PartitionFinder2 (Lanfear et al. 2017) integrated into PhyloSuite. Bayesian analysis was constructed using MrBayes v.3.2.6 (Ronquist et al. 2012) with four Markov chains for 1,000,000 generations, sampling every 100 generations. Standard deviation of split frequencies was controlled within 0.01 to ensure the convergence of the independent runs. The majority-rule consensus tree and estimation of the posterior probabilities (PP) were performed with the first 25% of samples discarded as burn-in.

**Table 1.** List of primers used in the study.

	Primer	Sequence	Reference
*rbcL*	ESRBCL1F	ATGTCACCACAAACGGAGACTAAAGC	Schuettpelz and Pryer (2007)
	ESRBCL1361R	TCAGGACTCCACTTACTAGCTTCACG	Schuettpelz and Pryer (2007)
*atpA*	ESATPF412F	GARCARGTTCGACAGCAAGT	Schuettpelz et al. (2006)
	ESTRNR46F	GTATAGGTTCRARTCCTATTGGACG	Schuettpelz et al. (2006)
*rps4*	RPS5*	ATGTCCCGTTATCGAGGACCT	Nadot et al. (1994)
	TRNS*	TACCGAGGGTTCGAATC	Souza-Chies et al. (1997)
*trnG-trnR*	TRNG1F^a^	GCGGGTATAGTTTAGTGGTAA	Korall et al. (2007)
	TRNR22R^a^	CTATCCATTAGACGATGGACG	Korall et al. (2007)
*rbcL-accD*	RBCL1187F^a^	GGAACYTTGGGACATCCTTGG	Korall et al. (2007)
	ACCD816R^a^	CCATGATCGAATAAAGATTCAGC	Ebihara et al. (2003)

**Table 2.** Best nucleotide substitution model in phylogenetic analyses.

Partition names	MrBayes	Sites
*atpA*, *trnG-trnR*	GTR	2 098
*rbcL*, *rps4*	GTR+G	1 279
*rbcL-accD*	GTR+I+G	806

## ﻿Results and discussion

### ﻿Morphological comparison

**Figure 1.**
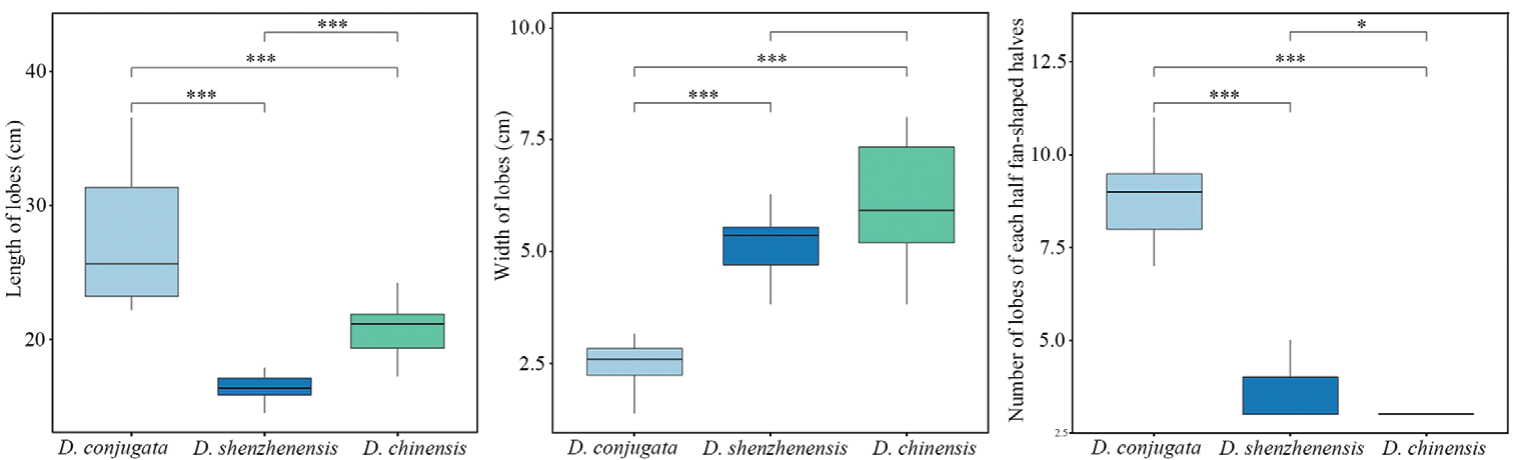
Boxplot (median and interquartile values) for the length of lobes (left), the width of lobes (center), and the number of lobes of each half of the fan-shaped fronds (right) amongst *Dipterisconjugata*, *D.shenzhenensis* and *D.chinensis*. Significant differences between species are represented with * (P < 0.05), ** (P < 0.001), and *** (P < 0.0001).

**Figure 2.**
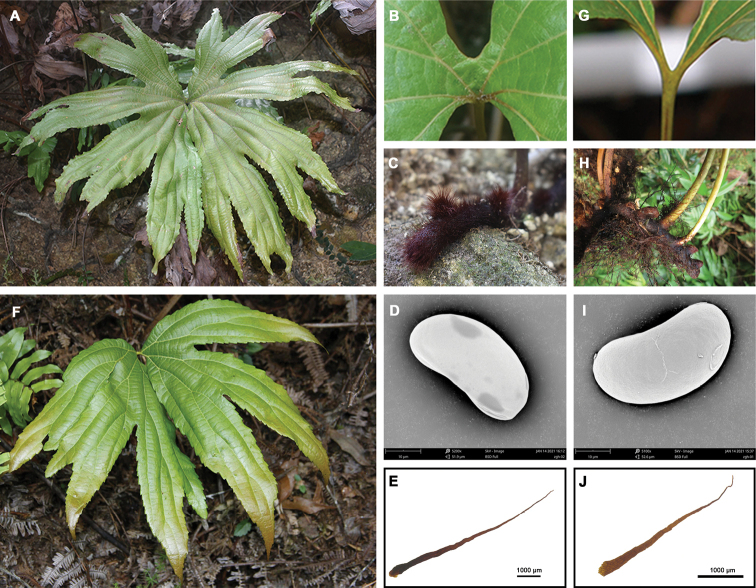
Morphological features of *Dipterisshenzhenensis* Y.H.Yan & Z.Y.Wei (A–E) and *D.chinensis* (F–J). **A** fan-shaped frond of *D.shenzhenensis***B** the frond base with broad wing of *D.shenzhenensis***C** rhizome of *D.shenzhenensis***D** spore of *D.shenzhenensis***E** rhizome scale of *D.shenzhenensis***F** fan-shaped frond of *D.chinensis***G** the frond base without wing of *D.chinensis***H** rhizome of *D.chinensis***I** spore of *D.chinensis***J** rhizome scale of *D.chinensis*.

**Figure 3.**
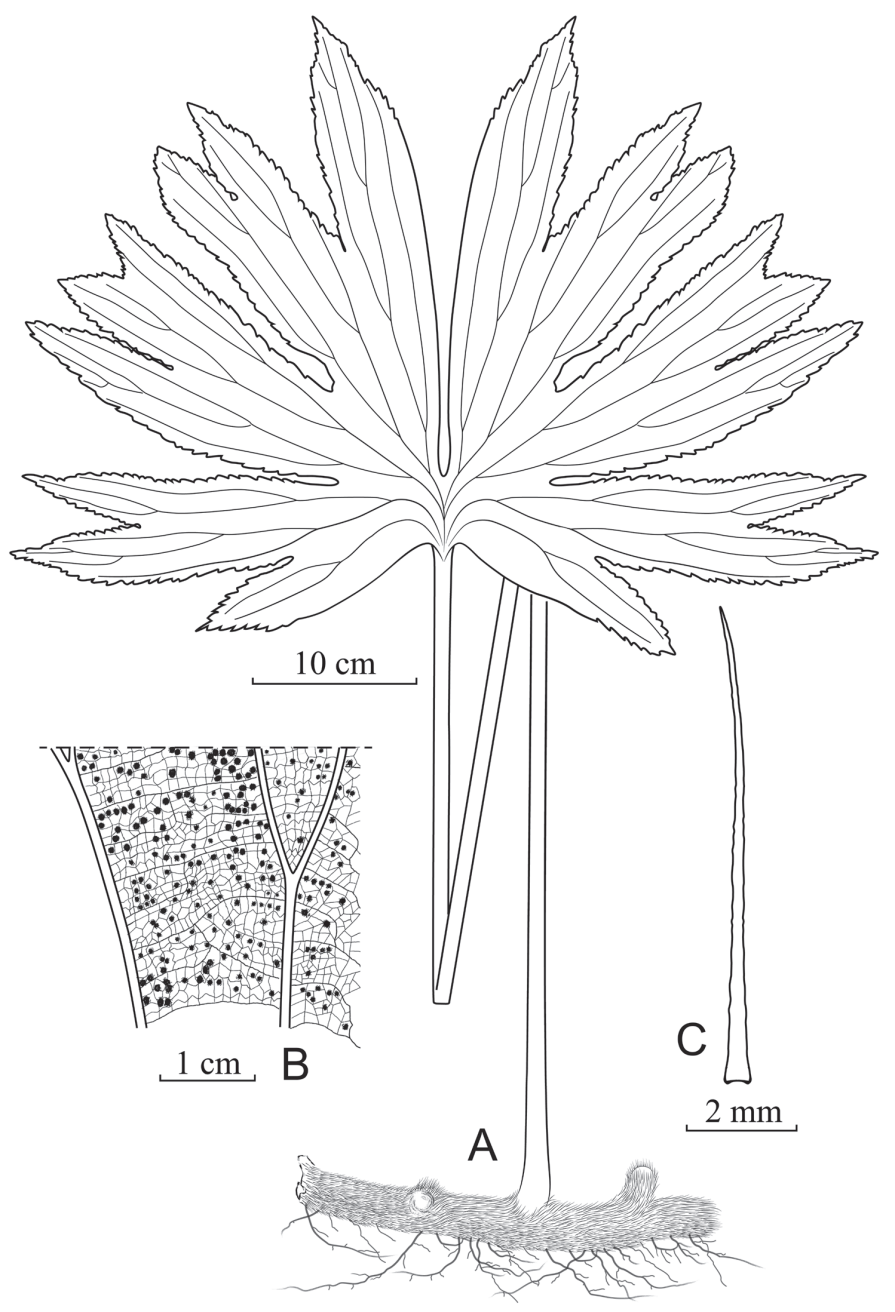
*Dipterisshenzhenensis* Y.H.Yan & Z.Y.Wei **A** habit **B** details of a lamina showing the venation and the distribution of sori **C** rhizome scale showing the profile and length (drawn by Zuo-Ying Wei & Li-Jun Chen, based on the type material at SZG).

*Dipterisshenzhenensis* has been confused with *D.conjugata* and *D.chinensis* because of similar gross morphology. This is especially true of dried herbarium specimens. Most specimens of *D.shenzhenensis* were formerly identified as *D.chinensis* in herbaria because of the presence in similar fronds morphology. We studied most online specimens of these three species and conducted quantitative trait statistical analysis. The result showed that *D.conjugata* displayed significant differences compared to *D.shenzhenensis* and *D.chinensis* in the length of lobes (LL) (*P* < 0.0001), the width of lobes (WL) (*P* < 0.0001), and the number of lobes for each half of the fan-shaped fronds (NL) (*P* < 0.0001) (Fig. 1). *Dipterisshenzhenensis* and *D.chinensis* were indistinguishable from the WL (*P* > 0.05), with the significant difference being in the LL (*P* < 0.0001) and the NL (*P* < 0.05) (Fig. 1). The former was also readily distinguished from the latter by having stiffer rhizome scales and castaneous stipe, as well as being pale abaxially (Fig. 2, Table 3). Micromorphological comparison indicated that the rhizome scale length of *D.shenzhenensis* was twice that of *D.chinensis* (Fig. 2). Most notably, the two fan-shaped fronds of *D.shenzhenensis* were connected by broad wings at the base in contrast to these of *D.conjugata*, *D.chinensis*, and other species in *Dipteris* (Figs 2 and 3).

**Table 3.** Comparisons of morphological characters and elevational range of *Dipterisshenzhenensis* and *D.chinensis*.

Character	*D.shenzhenensis*	*D.chinensis*
Rhizome	Long and creeping	Creeping
Rhizome scales	Dark brown to black, 6.8–8.0 × 0.06–0.27 mm	Brown, 3.74–4.00 × 0.04–0.26 mm
Fronds	Abaxially pale; base with broad wings	Abaxially green; base without wings
Lobes	4–6 × 14.5–18.0 cm	4–8 × 17–23 cm
Stipe	Castaneous, 40–85 cm	Stramineous to brown, 25–50 cm
Spores	18.5–19 × 37.5–39 μm	21.5– 25.5× 32.5–39 μm
Elevation	70–200 m	500–2100 m

### ﻿Phylogenetic analyses

**Figure 4.**
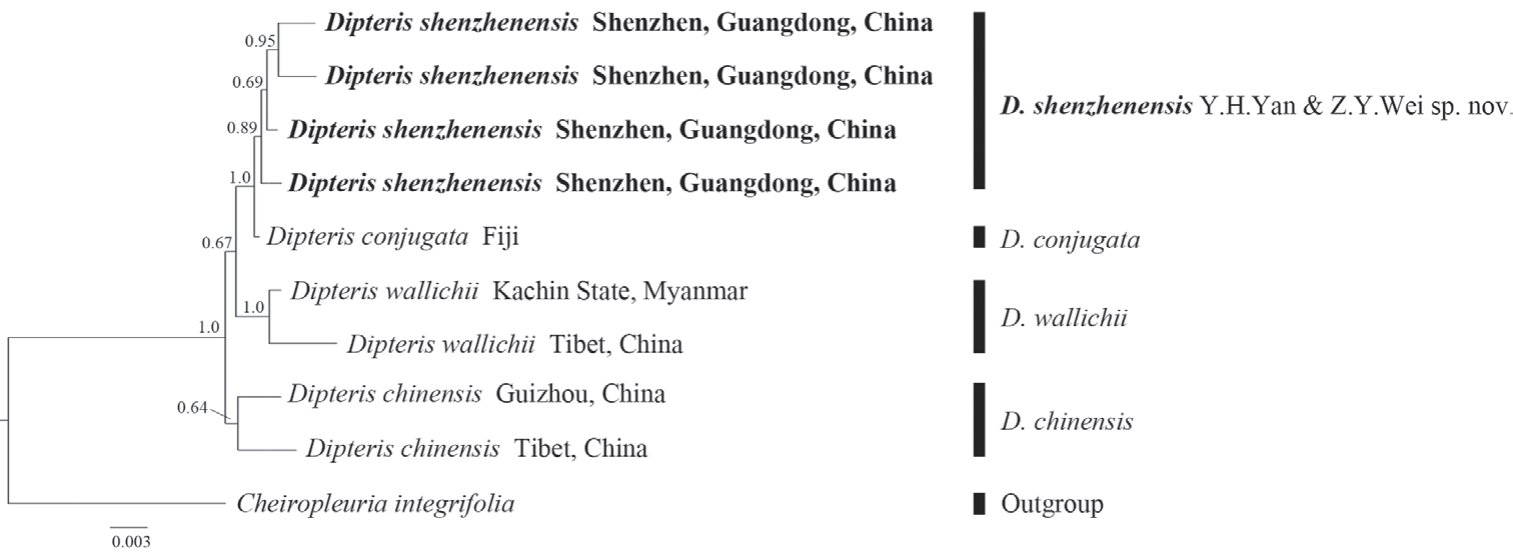
The phylogenetic tree inferred by the Bayesian inference with posterior probabilities above the branches.

To further determine the relationships among the three species, we conducted Bayesian analysis using the five chloroplast gene regions (*rbcL*, *atpA*, *rps4*, *rbcL-accD*, and *trnG-trnR*). With the *Cheiropleuriaintegrifolia* as outgroup, the phylogram showed that *Dipteris* can be classified into four well-supported clades. *Dipterisshenzhenensis* was typically well supported as monophyletic and strongly supported as sister to *D.conjugata* (PP = 1.0) (Fig. 4). Although *D.shenzhenensis* has been misidentified as *D.chinensis*, the relationship between the two species was not close. In addition, because there were missing data form many samples, the several clades showed a relatively low resolution in Bayesian phylogenetic analyses. We will, in future, use more molecular markers or utilize high-throughput sequencing to obtain a better topology with resolution.

Overall, based on the above morphological comparison and molecular phylogenetic analyses, *D.shenzhenensis* is clearly different from *D.conjugata* and *D.chinensis*. We therefore here describe *D.shenzhenensis* as a new species.

### ﻿Taxonomic treatment

#### ﻿ Dipteris shenzhenensis

Taxon classificationPlantaeGleichenialesDipteridaceae

Y.H.Yan & Z.Y.Wei sp. nov.

6B002A23-1C5A-5A46-A072-1C65B55DD2F6

urn:lsid:ipni.org:names:77234195-1

Figs 2 and 3

##### Diagnosis.

The new species is similar to *D.chinensis*, but differs in rhizome scales being longer (6.8–8.0 mm vs. 3.74–4.00 mm), in the base and color of fronds (base with broad wings, abaxially pale vs. base without wings, abaxially green), and in stipe color (castaneous vs. stramineous to brown).

##### Type.

China. Guangdong Province: Shenzhen City, Mt. Qiniangshan, elev. ca. 82 m, 16 August 2020, *Y. H. Yan* et al. *YYH15638* (***holotype***: SZG!; ***isotype***: NOCC!)

##### Description.

**Plants.** terrestrial on rocks, 0.5–1.0 m tall. **Rhizome.** long-creeping, ca. 1 cm in diam., densely scaly. **Rhizome scales.** spreading, dark brown to black, stiff, margin almost entire, 6.8–8.0 × 0.09–0.27 mm, lanceolate, apex long acuminate, acumen up to 2–3 mm long; **Stipe.** glabrous except at the very base, castaneous, 30–85 cm. **Fronds.** slightly funnel-shaped, divided into 2 fan-shaped fronds, each half deeply divided into 4 to 5 unequal lobes, lobes shallowly divided one or more times, with 8–10 ultimate lobes in each half of lamina, abaxially glabrous and pale; base with broad wings; venation reticulate, visible on both surfaces, prominent abaxially. **Lobes.** margins serrate, apices acute, 4.5–12.0 × 10.5–17.0 cm, reticulated venation network. **Spores.** spreading, monolete, 18.5–19.0 × 37.5–39.0 μm, glabrous.

##### Distribution and habitat.

So far only known from Shenzhen City, Guangdong Province, southern China. It is distributed in Mt. Qiniangshan, growing on rocks at elevation of 70–200 m in evergreen broad-leaf forest.

##### Chinese name.

Shen-zhen-shuang-shan-jue (深圳双扇蕨).

##### Etymology.

*Dipterisshenzhenensis* was discovered in the City of Shenzhen located in Guangdong Province, southern China. The specific epithet, therefore, is from this city name.

##### Conservation status.

*Dipterisshenzhenensis* is currently found in only one location in Mt. Qiniangshan, Shenzhen City, Guangdong Province, southern China. The predicted Area of Occupancy (AOO) for the species is no more than 5,000 m^2^. This species prefers to grow in low and opening mountain areas and is very likely to experience human disturbance. Over the past 20 years, the authors have observed that *D.shenzhenensis* showed signs of decline with the recovery of macrophanerophytes in Mt. Qiniangshan. Following the International Union for Conservation of Nature (IUCN) Categories and Criteria (IUCN 2019), we regard the newly discovered *D.shenzhenensis* as of Critically Endangered (CR) (B1a; B2ab).

##### Additional specimens examined.

China. Guangdong Province, Shenzhen City, Mt. Qiniangshan, elev. ca. 90 m, 16 August 2020, *Y. H. Yan* et al. *YYH15637* (NOCC!); loc. cit., elev. ca. 200 m, 27 December 2003, *Y. H. Yan 1937* (HUST!); loc. cit., elev. ca. 150 m, 8 November 2002, *Y. H. Yan 885* (HUST!); loc. cit., 17 August 2002, *S. Z. Zhang* et al. *011036-A1* (SZG, photo!); loc. cit., elev. ca. 75.38 m, 18 November 2015, *L. Jiang* & *Y. P. Chen JL00328* (KUN, photo!); loc. cit., elev. ca. 70 m, 22 February 2003, *S. Z. Zhang* et al. *012037-A* (SZG, photo!); loc. cit., elev. ca. 70 m, 22 February 2003, *S. Z. Zhang* et al. *012037-B* (SZG, photo!); loc. cit., 31 March 2000, *F. W. Xing* & *Y. X. Zhang 12374* (IBSC, photo!). s. coll. 0685742 (IBSC, photo!)

## Supplementary Material

XML Treatment for Dipteris shenzhenensis

## Supplementary materials

Supplementary material 1 Table S1. List of species and GenBank accession numbers used in the present study Data type molecular data Explanation note Dash (—) indicates unavailable data. https://binary.pensoft.net/file/620829This dataset is made available under the Open Database License (http://opendatacommons.org/licenses/odbl/1.0/). The Open Database License (ODbL) is a license agreement intended to allow users to freely share, modify, and use this Dataset while maintaining this same freedom for others, provided that the original source and author(s) are credited.Zuo-Ying Wei, Yu-Feng Gu, Zeng-Qiang Xia, Li-Jun Chen, Ting Wang, Shou-Zhou Zhang, Guo-Hua Zhao, Jian-Bing Chen, Jian-Guo Cao, Yue-Hong Yan

Supplementary material 2 Table S2. Specimen information used for morphological comparison Data type species data https://binary.pensoft.net/file/620830This dataset is made available under the Open Database License (http://opendatacommons.org/licenses/odbl/1.0/). The Open Database License (ODbL) is a license agreement intended to allow users to freely share, modify, and use this Dataset while maintaining this same freedom for others, provided that the original source and author(s) are credited.Zuo-Ying Wei, Yu-Feng Gu, Zeng-Qiang Xia, Li-Jun Chen, Ting Wang, Shou-Zhou Zhang, Guo-Hua Zhao, Jian-Bing Chen, Jian-Guo Cao, Yue-Hong Yan
