# Supplementary material for: ﻿Dipterisshenzhenensis, a new endangered species of Dipteridaceae from Shenzhen, southern China
Source: PhytoKeys. 2021 Dec 9;186:111–20. doi: 10.3897/phytokeys.186.73739 (PMC8677712; doi:10.3897/phytokeys.186.73739)
Supplement: Supplementary material 1 — Table S1. List of species and GenBank accession numbers used in the present study [file phytokeys-186-111-s001.docx]

**Table S1.** List of species and GenBank accession numbers used in the present study. Dash (—) indicates unavailable data.

| **Species** | **Locations** | **Voucher** | **GenBank accession number** | | | | | **Reference** |
| --- | --- | --- | --- | --- | --- | --- | --- | --- |
|  |  |  | ***rbcL*** | **atpA** | ***rps4*** | ***rbcL-accD*** | ***trnG-trnR*** |  |
| *Dipteris shenzhenensis* | Guangdong, China | YYH15636 | MZ365236 | MZ365242 | MZ365248 | MZ365256 | MZ365263 |  |
| *Dipteris shenzhenensis* | Guangdong, China | YYH15637 | MZ365237 | MZ365243 | MZ365249 | MZ365257 | MZ365264 |  |
| *Dipteris shenzhenensis* | Guangdong, China | YYH15638 | MZ365238 | MZ365244 | MZ365250 | MZ365258 | MZ365265 |  |
| *Dipteris shenzhenensis* | Guangdong, China | YYH15638-1 | MZ365239 | MZ365245 | MZ365251 | MZ365259 | MZ365266 |  |
| *Dipteris chinensis* | Guizhou, China | GZ7498 | MZ365233 | MZ365241 | — | MZ365253 | MZ365262 |  |
| *Dipteris chinensis* | Tibet, China | MTDEG-381 | MZ365235 | MZ365240 | MZ365247 | MZ365255 | MZ365261 |  |
| *Dipteris wallichii* | Tibet, China | CPG64005 | MZ365234 | — | — | MZ365254 | MZ365260 |  |
| *Dipteris wallichii* | Putao, Kachin State, Myanmar | CPG37192 | MZ365232 | — | MZ365246 | MZ365252 | — |  |
| *Dipteris conjugata* | Fiji | J. Game 98/106 | EF588692 | EF588670 | AY612658 | EF588736 | EF588779 | Metzgar et al. (2008) |
| *Cheiropleuria integrifolia* | Malaysia | Suzuki 00-01 | AB042569 | EF463661 | AY612654 | — | — | Schuettpelz and Pryer (2007) |
