# Supplementary material for: ﻿Dipterisshenzhenensis, a new endangered species of Dipteridaceae from Shenzhen, southern China
Source: PhytoKeys. 2021 Dec 9;186:111–20. doi: 10.3897/phytokeys.186.73739 (PMC8677712; doi:10.3897/phytokeys.186.73739)
Supplement: Supplementary material 2 — Table S2. Specimen information used for morphological comparison [file phytokeys-186-111-s002.docx]

**Table S2.** Specimen information used for morphological comparison.

| **Species** | **Location** | **Voucher** | **Herbarium** |
| --- | --- | --- | --- |
| *Dipteris conjugata* | Iriomote Island, Japan | Sather, D. 6223 | University of North Caroline Herbarium (NCU) |
| *D. conjugata* | Taipei, China | Boufford, D. E. 19446 | University of North Caroline Herbarium (NCU) |
| *D. conjugata* | Iriomote Island, Japan | Sather, D. 6237 | University of North Caroline Herbarium (NCU) |
| *D. conjugata* | Tjibodas, Java | A.G.S. s.n. | Chrysler Herbarium (CHRB) |
| *D. conjugata* | Luzon, Baguio | Steiner, N. L. 173 | Philippine National Herbarium (PNH) |
| *D. conjugata* | Fiji, Suva | Fawcett, S. & Game, J. C. 599 | University of Vermont, Pringle Herbarium (VT) |
| *D. conjugata* | Indonesia | Rosenstock 7555 | Naturalis Biodiversity Centre, formerly Leiden University |
| *D. conjugata* | Luzon, Laguna | s. coll. MT00200637 | Marie-Victorin Herbarium (MT) |
| *D. conjugata* | Philippine | Wenzel, C. A. 785 | The Field Museum (F) |
| *D. conjugate* | Philippine | Merrill, E. D. 3228 | United States National Herbarium (NMNH) |
| *D. conjugata* | Philippines | s. coll. 01419762 | United States National Herbarium (NMNH) |
| *D. conjugata* | Sarawak Museum | Native 2215 | United States National Herbarium (NMNH) |
| *D. conjugata* | Philippines | Bamos, M., Edano G. 30735 | New York Botanical Garden (Bronx) |
| *D. conjugata* | Taipei, China | Ando Y., Tateishi Y., Watanabe M. 761 | Herbarium, Kunming Institute of Botany, Chinese Academy of Sciences (KUN) |
| *D. conjugata* | Luzon, Bataan | P.P.E. L1.z 2045 | University of Michigan Herbarium (MICH) |
| *Dipteris shenzhenensis* | Guangdong, China | Y. H. Yan 885 | Herbarium of Hunan University of Science and Technology (HUST) |
| *D. shenzhenensis* | Guangdong, China | L. Jiang, Y. P. Chen JL00328 | Herbarium, Kunming Institute of Botany, Chinese Academy of Sciences (KUN) |
| *D. shenzhenensis* | China | s. coll. 0685742 | Herbarium, South China Botanical Garden, Chinese Academy of Sciences (IBSC) |
| *D. shenzhenensis* | Guangdong, China | Y. H. Yan et al. YYH15636 | National Orchid Conservation center of China (NOCC) |
| *D. shenzhenensis* | Guangdong, China | Y. H. Yan et al. YYH15637 | National Orchid Conservation center of China (NOCC) |
| *D. shenzhenensis* | Guangdong, China | Y. H. Yan et al. YYH15638 | Herbarium, Fairy Lake Botanical Garden, Shenzhen & Chinese Academy of Sciences (SZG) |
| *D. shenzhenensis* | Guangdong, China | S. Z. Zhang et al. 011036-A1 | Herbarium, Fairy Lake Botanical Garden, Shenzhen & Chinese Academy of Sciences (SZG) |
| *D. shenzhenensis* | Guangdong, China | S. Z. Zhang et al. 011037-A | Herbarium, Fairy Lake Botanical Garden, Shenzhen & Chinese Academy of Sciences (SZG) |
| *D. shenzhenensis* | Guangdong, China | S. Z. Zhang et al. 011037-B | Herbarium, Fairy Lake Botanical Garden, Shenzhen & Chinese Academy of Sciences (SZG) |
| *Dipteris chinensis* | Yunnan, China | Henry, A. 9041B | New York Botanical Garden (Bronx) |
| *D. chinensis* | Guizhou, China | Cavalerie P. J. 341 | Royal Botanic Garden Edinburgh (E) |
| *D. chinensis* | Guizhou, China | Cavalerie, H. 371 | Natural History Museum (BM) |
| *D. chinensis* | Guizhou, China | Cavalerie P. J. 341 | Herbier Muséum Paris (P) |
| *D. chinensis* | Guizhou, China | Cavalerie, C. 341 | Royal Botanic Gardens, Kew (K) |
| *D. chinensis* | Guizhou, China | L. Yang 92-602 | Guizhou Normal University (GNUG) |
| *D. chinensis* | Guangxi, China | L. Wu & Y. Tong 3517 | Beijing Normal University (BNU) |
| *D. chinensis* | Yunnan, China | s. coll. 119 | Herbarium, Kunming Institute of Botany, Chinese Academy of Sciences (KUN) |
| *D. chinensis* | Guizhou, China | Z.Y. Guo 7498 | Herbarium, Institute of Botany, Academia Sinica |
| *D. chinensis* | Chongqing, China | Z. Y. Liu 992627 | Herbarium, Chongqing Institute of Medicinal Plant Cultivation (IMC) |
